# Subcutaneous panniculitis-like T-cell lymphoma complicated by hemophagocytic syndrome with significant response to pralatrexate: A case report

**DOI:** 10.1016/j.jdcr.2025.07.027

**Published:** 2025-08-22

**Authors:** Jason Chen, Vincent Liu, Eric Mou

**Affiliations:** a University of Iowa Carver College of Medicine, Iowa City, Iowa; b Departments of Dermatology and Pathology, University of Iowa Carver College of Medicine, Iowa City, Iowa; c Division of Hematology, Oncology, and Blood & Marrow Transplant, University of Iowa Carver College of Medicine, Iowa City, Iowa

**Keywords:** cytotoxic, hemophagocytic syndrome, pralatrexate, subcutaneous panniculitis-like T-cell lymphoma

## Introduction

Subcutaneous panniculitis-like T-cell lymphoma (SPTCL) is a rare subtype of primary cutaneous lymphoma with a median age of presentation of 36 years.^1^ SPTCL is slightly more common in females, frequently diagnosed in patients with autoimmune conditions, and pathologically characterized by a dermal and pannicular infiltrate resembling panniculitis, but composed of CD8-expressing cytotoxic T-cells exhibiting an αβ T-cell receptor phenotype.^2^^,^^3^ SPTCL typically presents as multifocal subcutaneous nodules with overlying erythema and, in contrast to primary cutaneous gamma-delta T-cell lymphoma, exhibits an indolent disease course with a favorable outlook. However, 15% to 20% of cases are accompanied by hemophagocytic syndrome (HPS) and demonstrate a markedly poorer prognosis.^1^ While the pathophysiology of SPTCL remains uncertain, biallelic germline coding variants in the hepatitis A virus-cellular receptor 2 (*HAVCR2*) gene, resulting in decreased expression of the immune checkpoint T-cell immunoglobulin mucin (TIM3), are increasingly implicated in driving the immune dysregulation underlying this disease and are associated with an increased risk of HPS.^4^ Due to both its rarity and heterogeneity, the optimal treatment for SPTCL is undefined, with therapeutic options ranging from low-intensity immunomodulation to polychemotherapy, often tailored to disease phenotype. Here, we report a case of SPTCL complicated by severe HPS and disseminated intravascular coagulation (DIC), in which intensive therapy was contraindicated, successfully treated with pralatrexate.

## Case report

A 54-year-old African American female presented with new-onset seizures, 20 pounds of unintentional weight loss, and nodular skin lesions over both arms. Magnetic resonance imaging of the brain was normal, and a lumbar puncture was negative for infection or malignancy. Computed tomography scans of the chest, abdomen, and pelvis revealed multiple 1-2 cm bilateral axillary and mediastinal lymph nodes. A skin biopsy showed nonspecific chronic granulomatous dermatitis and panniculitis. Prednisone 20 mg daily was begun empirically. The patient was then referred to our dermatology clinic for further care.

**Fig 1:**
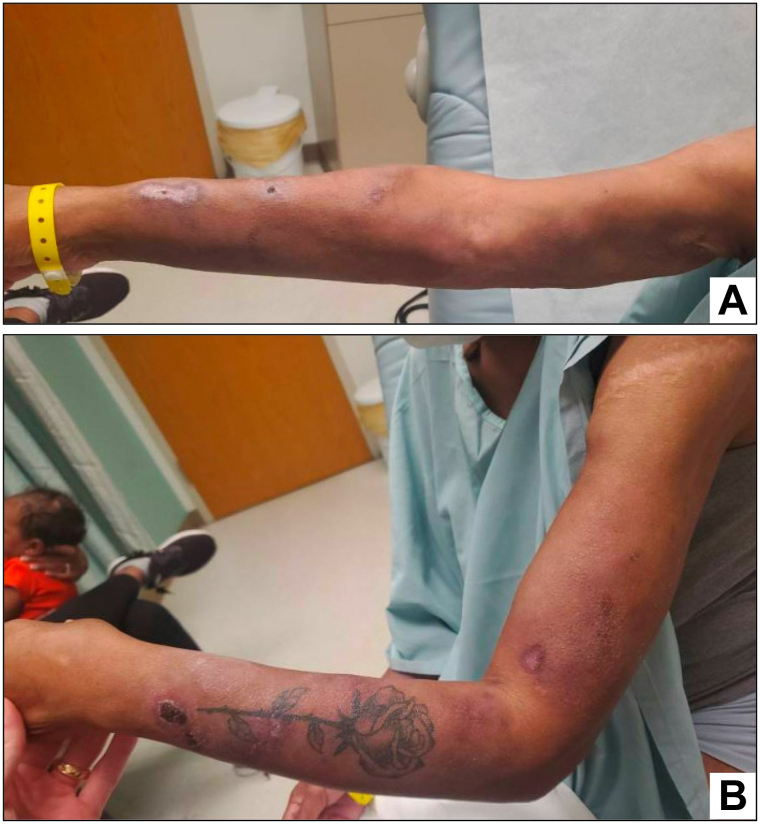
Skin appearance of bilateral arms at time of initial dermatology evaluation. Right **(A)** and left **(B)** arms showing multifocal erythematous dermal nodules with variable overlying hemorrhagic crust and accompanying lipoatrophy.

**Fig 2:**
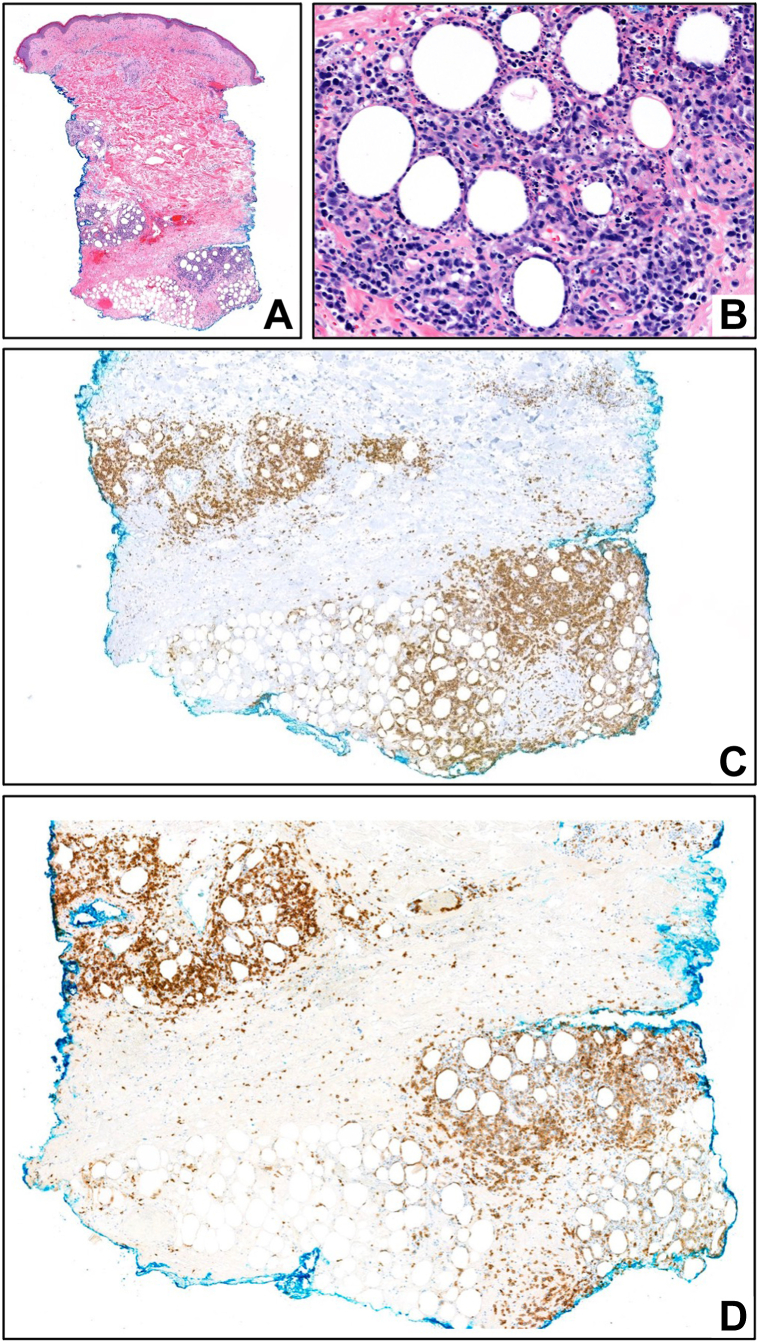
Skin punch biopsy of right arm. **A,** Scanning magnification showing a lymphocytic pannicular infiltrate [hematoxylin and eosin (H&E); 20×]; **(B)** adipocytic rimming by atypical lymphocytes [H&E; 200×]. Immunohistochemical stains for CD8 [CD8; 50×] **(C)** and T-cell receptor (TCR)-beta-F1 [TCR-beta-F1; 60×] **(D)** highlight lobular pannicular lymphocytic infiltrate.

Upon initial evaluation, persistent dermal nodules with scattered erythematous papules and variable overlying hemorrhagic crust involving the bilateral arms and torso, and palpable bilateral axillary lymphadenopathy were observed (Fig 1). Skin biopsies of the right arm and abdomen showed an atypical pannicular cytotoxic T-lymphocytic infiltrate positive for CD8 and T-cell receptor-beta-F1, with areas of adipocytic rimming, consistent with SPTCL (Fig 2, *A*-*D*). Positron-emission tomography/computed tomography scan showed extensive subcutaneous involvement and bilateral axillary nodes measuring 1-2 cm. Core needle biopsy of a right axillary lymph node revealed reactive changes with no evidence of malignancy.

**Fig 3:**
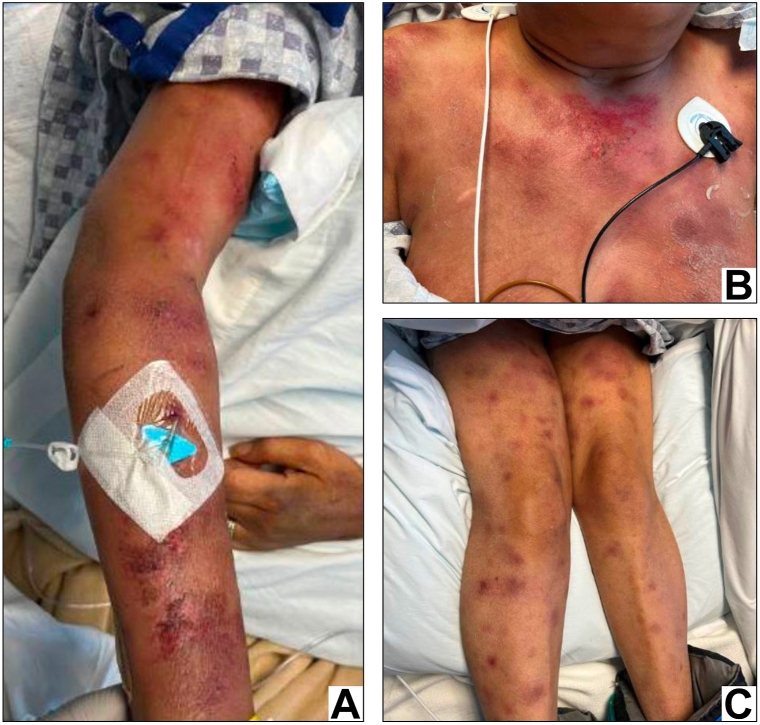
Skin appearance at time of acute hospitalization. Right arm **(A),** anterior chest **(B),** and bilateral lower extremities **(C)** show multifocal dermal nodules with variably erosive changes and overlying hemorrhagic crust.

Two weeks later, the patient presented to the emergency department with a grand mal seizure and encephalopathy. A magnetic resonance imaging of the brain showed acute right thalamic intraparenchymal hemorrhage. The patient’s skin lesions had significantly worsened, with numerous dermal nodules and erythematous papules distributed over her chest and bilateral upper and lower extremities (Fig 3). Laboratory studies revealed new anemia (11.2 g/dL; ref: 13.2-17.7 g/dL), low platelets (117 k/mm^3^, 150-400 k/mm^3^), elevations in alanine aminotransferase (85 U/L, 0-41 U/L), aspartate aminotransferase (205 U/L, 0-40 U/L), lactate dehydrogenase (1601 U/L, 135-225 U/L), and ferritin (59,259 ng/mL, 30-400 ng/mL), undetectably low fibrinogen, and prolonged international normalized ratio (>10.0) and partial thromboplastin time (>150 seconds, 22-31 seconds). These findings were consistent with hyperinflammation and DIC secondary to HPS.

**Fig 4:**
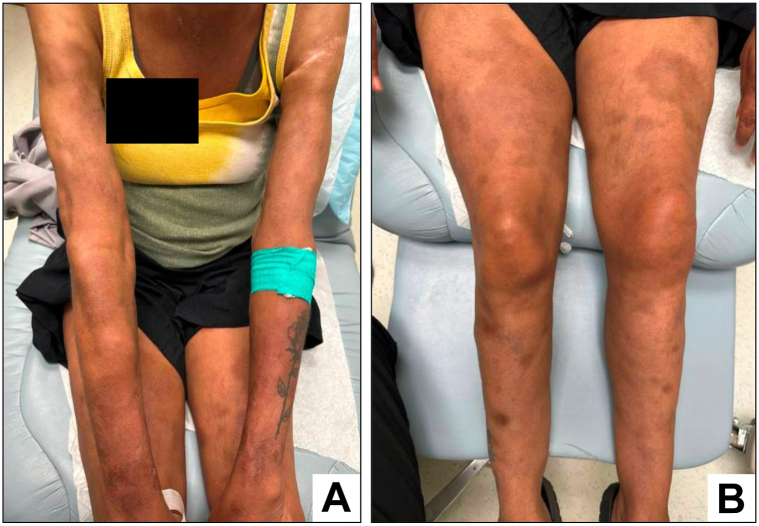
Skin appearance following pralatrexate therapy. Bilateral arms **(A)** and bilateral lower extremities **(B)** with near-complete resolution of multifocal dermal nodules and residual postinflammatory hyperpigmentation and lipoatrophy.

HPS-directed therapy was begun with high-dose corticosteroids, anakinra, and intravenous immunoglobulin. At this time, the patient was severely deconditioned, persistently encephalopathic and coagulopathic, and afflicted by multiple infections, including polymicrobial bacteremia and candidal fungemia. Following multiple goals of care discussions with family, SPTCL-directed therapy was started with pralatrexate 15 mg/m2 every 1-2 weeks. Over the following month, the patient gradually improved, showing a good partial response in skin lesions and gradual resolution of encephalopathy, coagulopathy, and hyperferritinemia. Pralatrexate was continued for another 3 months with further improvement (Fig 4), after which time the subtle appearance of new lesions prompted a switch to cyclosporine. Following 18 months of cyclosporine (75-200 mg daily), and intermittent intralesional corticosteroid injections, the patient’s disease burden remains minimal and stable, her performance status is excellent, and consolidative allogenic stem cell transplantation is planned.

## Discussion

Recognizing that SPTCL exhibits heterogeneity in its clinical presentation, course, and disease cadence, Willemze et al asserted that patients do not uniformly benefit from conventionally aggressive chemotherapy.^1^ Less intensive therapies like systemic corticosteroids or immunosuppressive agents, including cyclosporine or methotrexate, have been successfully used.^2^ While patients with concurrent HPS are frequently considered for more intensive treatment and subsequent consideration of allogeneic stem cell transplantation in order to mitigate the poor outcomes associated with this phenomenon,^5^ selected patients may similarly undergo initial treatment with less intensive therapies such as corticosteroids, methotrexate, and/or cyclosporine A.^6^^,^^7^

In our patient’s case, her florid presentation with severe DIC and hyperinflammation resulted in profound debility, rendering her ineligible for the more intensive treatment approaches typically considered in this situation. Ultimately, pralatrexate as a single agent was selected in hopes of balancing the competing risks of lymphoma-directed efficacy and treatment-related toxicity, owing to prior description of its efficacy in the treatment of SPTCL with HPS.^8^ A recent retrospective study including 22 patients with SPTCL treated with contemporary therapies, 68% of whom exhibited clinical suspicion for HPS, showed that alongside combination chemotherapy, pralatrexate represented the most effective treatment, including successfully serving as a bridge to allogeneic stem cell transplantation in patients with concurrent HPS.^9^ Of note, given the increasingly recognized association between germline *HAVCR2* aberrations and risk of HPS in patients with SPTCL, detecting *HAVCR2* coding variants could aid in the early recognition of patients at increased risk for aggressive evolution of disease.^10^ In summary, the critical illness, hemorrhagic complications, and numerous infections experienced by our patient are illustrative of the broad spectrum of clinical manifestations that may accompany SPTCL, underscoring the importance of meticulous vigilance in caring for this patient population. Pralatrexate can be effective in treating SPTCL and offers an additional treatment option in the appropriate clinical context. Further investigation into the genetic underpinnings of SPTCL is essential to inform optimal prognostication and therapy.

## Conflicts of interest

None disclosed.
